# Impact of geriatric co-management on outcomes in hospitalised cardiology patients aged 85 and over

**DOI:** 10.1007/s12471-023-01806-y

**Published:** 2023-08-31

**Authors:** Renee C. M. A. Raijmann, Huiberdina L. Koek, Marielle H. Emmelot-Vonk, Joost G. E. Swaving, Willem R. P. Agema, Angèle P. M. Kerckhoffs, Carolina J. P. W. Keijsers

**Affiliations:** 1 Department of Geriatrics, Jeroen Bosch Ziekenhuis, ’s-Hertogenbosch, The Netherlands; 2 Department of Geriatrics, UMC Utrecht, Utrecht, The Netherlands; 3 Medical School, Radboud University, Nijmegen, The Netherlands; 4 Department of Cardiology, Jeroen Bosch Ziekenhuis, ’s-Hertogenbosch, The Netherlands

**Keywords:** Geriatric co-management, Quality improvement, Heart diseases, Aged

## Abstract

**Objective:**

Cardiovascular disease and frailty are common among the population aged 85+. We hypothesised these patients might benefit from geriatric co-management, as has been shown in other frail patient populations. However, there is limited evidence supporting geriatric co-management in older, hospitalised cardiology patients.

**Methods:**

A retrospective cohort study was performed in a large teaching hospital in the Netherlands. We compared patients aged 85 and over admitted to the cardiology ward before (control group) and after the implementation of standard geriatric co-management (intervention group). Data on readmission, mortality, length of stay, number of consultations, delirium, and falls were analysed.

**Results:**

The data of 1163 patients were analysed (*n* = 542 control, *n* = 621 intervention). In the intervention group, 251 patients did not receive the intervention because of logistic reasons or the treating physician’s decision. Baseline characteristics were comparable in the intervention and control groups. Patients in the intervention group had a shorter length of stay (−1 day, *p* = 0.01) and were more often discharged to a geriatric rehabilitation facility (odds ratio [OR] 1.97, 95% confidence interval [CI] 1.10–3.54, *p* = 0.02) compared with the control patients. Other outcomes were not significantly different between the groups.

**Conclusions:**

After implementation of standard geriatric co-management for hospitalised cardiology patients aged 85 and over, the length of hospital stay shortened and the number of patients discharged to a geriatric rehabilitation facility increased. The adherence to geriatric team recommendations was high. Geriatric co-management would appear to optimise care for older hospitalised patients with cardiac disease.

## What’s new?

- Addressing frailty in the older cardiac patient population is an important step in further improving quality of care for this patient population.
- This study demonstrates that standard geriatric co-management in older patients with cardiac disease can reduce length of hospital stay without increasing hospital readmission rates
- Furthermore, more patients are discharged to geriatric rehabilitation facilities in the co-managed group, which demonstrates a greater effort to manage functional decline in the elderly after hospital admission.

## Introduction

The peak prevalence of chronic cardiovascular disease has shifted to the oldest old as a result of improvements in the acute management of cardiovascular disease [1]. Older patients with cardiac disease typically suffer from frailty as well [2]. The prevalence of frailty in patients with heart failure is 44.5% [3], and frail patients are at an increased risk of hospitalisation and death [4]. It has been shown that 20% of elderly patients admitted to a cardiology unit are readmitted and 10% die within 1 month of discharge [5].

Various cardiovascular societies have recognised the importance of addressing frailty in the elderly population [6, 7]. However, healthcare staff are often inadequately trained to identify the complex needs of frail patients [8]. Issues such as depression, risk of falls, and cognitive impairment often go unrecognised, increasing the risk of adverse health events [9]. For this reason, clinicians have advocated the integration of geriatric care into cardiovascular disease management.

Current evidence suggests standard geriatric co-management as an option for such care integration. In contrast to regular consultation, co-management gives geriatricians more influence on treatment decisions [10]. A Cochrane review from 2017 demonstrated that patients who had undergone a Comprehensive Geriatric Assessment (CGA) were more likely to live at home after hospital admission [11]. Another meta-analysis showed a reduced length of hospital stay (LOS), better functional status, and decreased complications in favour of patients co-managed by geriatricians [12]. The studies included in these reviews were conducted on surgical and general medicine wards. The only trial conducted on a cardiac ward with older frail patients found geriatric co-management to reduce the rate of complications and functional decline during and after hospital admission [13].

Despite the proposed role of geriatric care in the management of older cardiology patients, few studies have investigated the effectiveness of this approach. In this study, we investigated the effect of standard geriatric co-management in a patient population aged 85 and over with cardiac disease.

## Methods

### Study design and setting

**Fig. 1:**
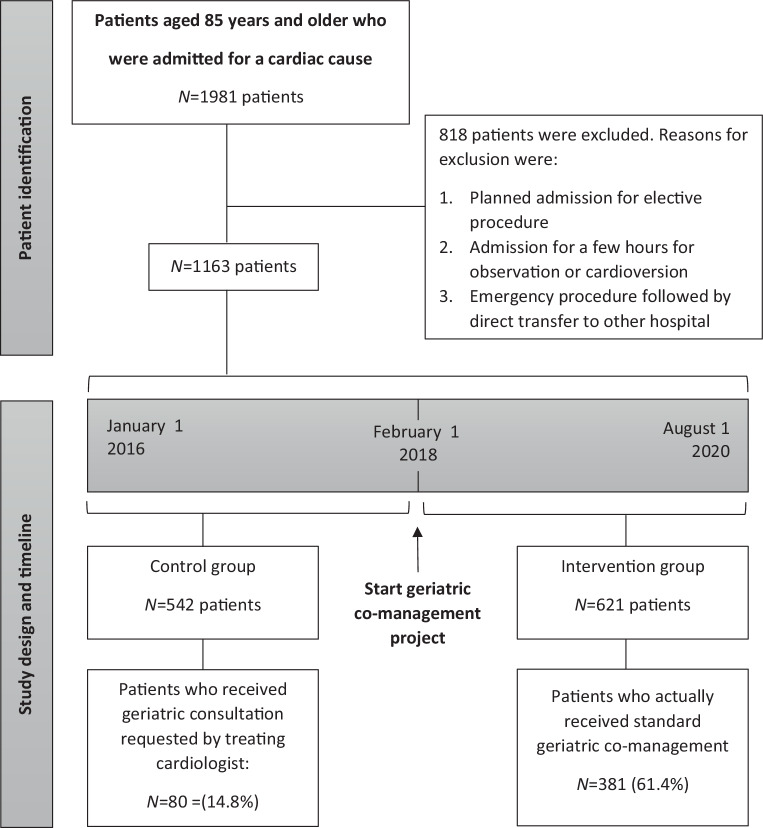
Study design and patient identification

The study had a single-centre, retrospective cohort design (Fig. 1). Different health outcomes were compared among patients who were admitted before (control group) and after the implementation of a geriatric co-management project on 1 February 2018 (intervention group). We retrospectively collected the data of patients admitted between 1 January 2016 and 1 August 2020. The study was performed in the Cardiac Department of the Jeroen Bosch Hospital, a large teaching hospital in the Netherlands. The STROBE (Strengthening the Reporting of Observational Studies in Epidemiology) guidelines were used in the design and reporting of this study.

### Inclusion criteria

All patients aged 85 and over who had an unplanned hospital admission to the cardiac ward for more than 24 h were eligible for inclusion. As the prevalence of frailty increases with age [14], an age cut-off of 85 years was used to include the highest number of frail patients and still make the project practically feasible.

### Intervention: standard geriatric co-management

**Fig. 2:**
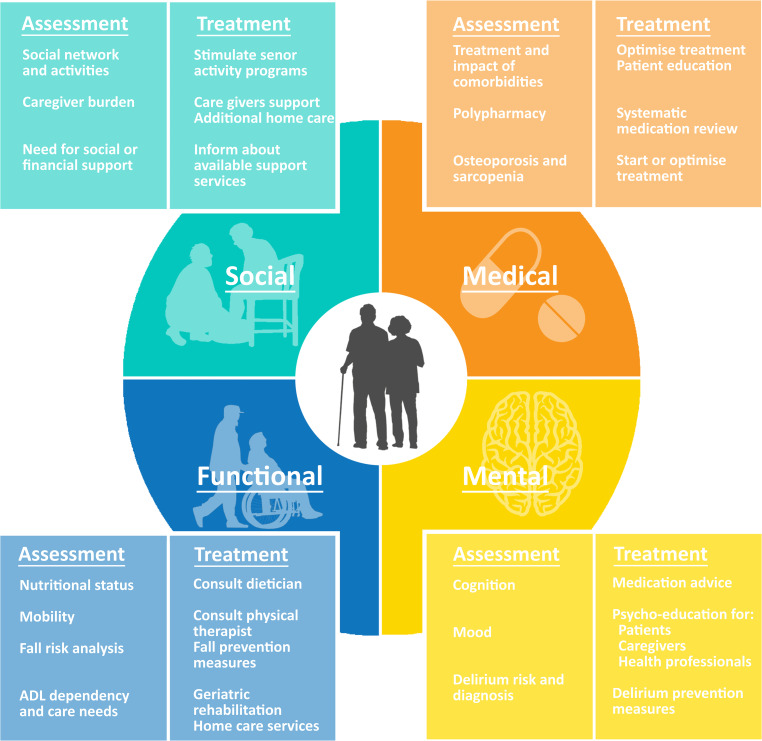
Comprehensive geriatric assessment

All eligible patients in the intervention group underwent a CGA (Fig. 2) within 48 h of admission performed by a geriatric physician and a geriatric nurse (the regular geriatric consultation team in our hospital). After the initial assessment, the geriatrician and treating cardiologist discussed tailored care for that patient. The geriatric team was involved in the patient’s further treatment, based on that patient’s needs. Cardiac nurses and a geriatric nurse met weekly to discuss admitted patients and their care.

The control group received usual care. If their treating physician requested a geriatric consultation during their admission, patients underwent the same CGA.

### Data collection

Electronic patient records were searched for eligible patients using the software program CTcue version 2.1.10 [15]. This software collects clinical data, using the previously mentioned inclusion criteria and time frame as search criteria.

Two researchers (R.R. and J.S.) manually searched medical records for additional data that could not be obtained with the software (e.g. residency). Patients who were falsely identified by the software were also excluded (see Fig. 1).

### Baseline characteristics

The following baseline characteristics were collected: general demographics, medical history, admission diagnosis, previous hospital admission in the last 12 months, and information obtained with the CGA (e.g. the number of medications used, number of falls over the last 6 months).

### Outcomes

The primary outcome was all-cause hospital readmission rate at 1, 6, and 12 months after the initial admission. Multiple time points were used to account for differences in follow-up time and multiple hospitalisations. The secondary outcomes were LOS, all-cause in-hospital mortality, all-cause mortality within 3 months of admission, number of interprofessional consultations, change in residency, or discharge to a rehabilitation facility. The number of complications, such as falls or delirium, were also recorded.

### Statistical analysis

Baseline characteristics were compared between groups using statistical tests (χ2-test, Fisher’s exact test, two-sample *t*-test and Mann-Whitney U test, where appropriate). All analyses were performed with an intention-to-treat approach to prevent selection bias. The primary outcome readmission rates and the incidence rate ratio were calculated using a Poisson regression model. These models were adjusted for baseline differences and risk factors for readmission.

For normally distributed secondary outcome variables odds ratios were calculated using logistic regression. The non-normal outcome variables, number of consultations and LOS, were first analysed using a Mann-Whitney U test. For the multivariate analysis of the number of consultations, a Poisson regression model was chosen. For the LOS data, a normal distribution was achieved through a square root transformation so a multiple linear regression model could be used. Furthermore, we attempted to reduce the influence of extreme outliers by truncating the data at 30 days. This means that the duration of hospitalisation for all patients hospitalised for longer than 30 days was changed to 30 days. All the multivariate models contained the variables age, number of comorbidities, and any baseline differences.

The data were analysed with the statistical package for social sciences (SPSS) version 25 [16]. Statistical inference was based on a *p*-value of 0.05.

### Sample size calculation

A power calculation was performed based on the incidence of our most important outcomes rehospitalisation, mortality, and LOS (β = 15%, α = 5%, two-sided test) [12, 17, 18]. For a clinically significant reduction in any of these outcomes, 1150 patients were needed.

### Ethical considerations

The regional Ethics Review Board (METC Brabant/20.435, #NW2020-78) deemed this study to fall outside the scope of the Dutch Law on Medical Research (WMO). The study was conducted in accordance with the Dutch Medical Treatment Contracts Act (WGBO) article 458 and the principles of the World Medical Association Declaration of Helsinki (2013). The physical and psychological integrity of the patients were not harmed in any way.

## Results

Of 1981 potentially eligible patients identified in our first digital search, 1163 remained after manual screening, of which 621 formed the intervention group (Fig. 1).

The patients’ baseline characteristics are described in Tab. 1. All variables were comparable except for the history of chronic heart disease.

**Table 1:** Comparison of baseline characteristics

		Overall (*n* = 1163)	Control group (*n* = 542)	Intervention group (*n* = 621)	*P*-value
Demographics	Age, median (IQR) ^†^	88 (4.0)	88 (4.0)	88 (4.0)	0.31
	Female, *n* (%)^‡^	695 (59.8)	329 (60.7)	366 (58.9)	0.55
Admission information	Admission diagnosis *n* (%)^‡^				0.30
	– Congestive heart failure	434 (37.3)	216 (39.9)	218 (35.1)	
	– Arrhythmia	230 (19.8)	96 (17.7)	134 (21.6)	
	– Acute coronary syndrome	214 (18.4)	102 (18.8)	112 (18.0)	
	– Acute decompensated heart failure	77 (6.6)	41 (7.6)	36 (5.8)	
	– Syncope	91 (7.8)	35 (6.5)	56 (9.0)	
	– Chest pain	71 (6.1)	30 (5.5)	41 (6.6)	
	– Cardiac arrest	10 (0.9)	5 (0.9)	5 (0.8)	
	– Other	36 (3.1)	17 (3.1)	19 (3.1)	
	Previous hospital admission in last 12 months *n* = 1128, (%)^‡^	272 (24.1)	129 (24.2)	143 (24.0)	0.94
Patient history	CCI score, median (IQR) ^‡^	6 (2.0)	6 (2.0)	6 (2.0)	0.05*
	Chronic heart disease, *n* (%)^‡^	858 (73.8)	428 (79.0)	430 (69.2)	< 0.01*
	– Chronic heart failure^‡^	356 (30.6)	185 (34.1)	171 (27.5)	0.02*
	– Ischaemic heart disease^‡^	368 (31.6)	194 (35.8)	174 (28.0)	< 0.01*
	– Heart valve dysfunction^‡^	301 (25.9)	157 (29.0)	144 (23.2)	0.03*
	– Arrhythmia^‡^	505 (43.4)	246 (45.4)	259 (41.7)	0.21
	– Other^‡^	19 (1.6)	8 (1.5)	12 (1.9)	0.49
	Peripheral vascular disease^‡^	151 (13.0)	80 (14.8)	71 (11.4)	0.10
	Neurological disorder^‡^	359 (30.9)	166 (30.6)	193 (31.1)	0.90
	Pulmonary disease^‡^	206 (17.7)	94 (17.3)	112 (18.0)	0.82
	Body mass index, mean (SD) ^§^	26.2 (4.6)	26.2 (4.5)	26.2 (4.6)	0.52
	Diabetes^‡^	280 (24.1)	134 (24.7)	146 (23.5)	0.63
	Chronic kidney disease^‡^	538 (46.3)	265 (48.9)	273 (44.0)	0.09
	Mean eGFR (SD) ^§^	52 (19.6)	49.7 (19.2)	54 (19.8)	0.46
	History of malignancy^‡^	271 (23.3)	113 (20.8)	158 (25.4)	0.07
	Mean haemoglobin (SD) ^§^	8.0 (2.4)	8.1 (3.3)	7.9 (1.1)	0.29
CGA components	Number of medications, median (IQR) ^†^	7 (5.0)	7 (4.0)	7 (5.0)	0.67
	SNAQ score, median (IQR) ^†^	0 (1.0)	0 (1.0)	0 (1.0)	0.54
	History of dementia^‡^	127 (10.9)	61 (11.3)	66 (10.6)	0.53
	History of delirium, *n* = 990 *n* (%)^‡^	181 (18.3)	79 (19.0)	103 (17.8)	0.68
	Fall history, *n* = 1030 *n* (%)^‡^	295 (28.6)	129 (29.8)	166 (27.8)	0.49
	Katz ADL score, *n* = 1056, median (IQR) ^†^	0 (2.0)	0 (2.0)	0 (3.0)	0.54
	Residential situation *n* = 1157 *n* (%)^‡^				0.74
	– Community	994 (85.9)	460 (85.5)	534 (86.3)	
	– Other	163 (14.1)	78 (14.5)	85 (13.7)	

*IQR* interquartile range, *SD* standard deviation, *eGFR* estimated glomerular filtration rate, *CGA* comprehensive geriatric assessment, *CCI* Charlson Comorbidity Index, *SNAQ* short nutritional assessment questionnaire [19], Katz ADL score; Katz index of activities of daily living score [20]

* *p*-value ≤ 0.05. For comparing characteristics, the Mann-Whitney U test †, chi-squared test ‡ and independent samples *t*-test § were used, where appropriate

### Implementation of geriatric co-management

Before the intervention, cardiologists consulted geriatricians concerning 14.8% of all admitted patients aged 85 and over. After intervention implementation, 61.4% of patients received geriatric co-management. If patients in the intervention group did not receive the intervention, this was because of logistic reasons (e.g. staff shortage in weekends) or because the treating physician decided against it. Patients in the intervention group who received geriatric co-management had a significantly higher prevalence of dementia (15.1% vs 8.1%), a previous fall or episode of delirium (22.9% vs 15.6% and 33.3% vs 25.1%, respectively), and a higher average Katz Index of Activities of Daily Living (Katz ADL) score [17] (median = 1, interquartile range [IQR] 3 vs median = 0, IQR 2) (all *p* < 0.01) when compared with the not co-managed intervention group patients.

### Hospital readmission rate

The total number of readmissions, readmission rates, and incidence rate ratio (IRR) are presented in Tab. 2. There were no significant differences between control and intervention groups in readmission rate after 1 month (IRR 0.87, 95% confidence interval [CI] 0.63–1.21), 6 months (IRR 1.07, 95% CI 0.86–1.34, *p* = 0.53), and 12 months (IRR 0.95, 95% CI 0.80–1.13, *p* = 0.56).

**Table 2:** Hospital readmission rates for the intervention group and the control group

		Cumulative number of readmissions	Cumulative number of person days	Readmission incidence rate per PY	IRR*	95% CI
Total follow-up	Intervention group	406	238,062	0.62	1.08	0.86–1.23
	Control group	619	364,931	0.62	1.00	
12-month follow-up	Intervention group	290	158,196	0.67	0.99	0.83–1.16
	Control group	279	141,796	0.72	1.00	
6‑month follow-up	Intervention group	186	88,691	0.77	1.08	0.87–1.35
	Control group	155	75,590	0.75	1.00	
1‑month follow-up	Intervention group	74	16,293	1.66	0.87	0.63–1.21
	Control group	77	13,808	2.04	1.00	

*PY* person years, *IRR* incidence rate ratio, *CI* confidence interval

*IRR is calculated in a multivariate model including age, previous hospital admission, history of chronic heart disease and Charlson Comorbidity Index.

### Secondary outcomes

Patients in the intervention group had a significantly shorter hospital stay than patients in the control group in univariate (*r* *=* *0.08; p* = 0.01) and multivariate linear regression analyses (*p* = 0.04). Furthermore, more intervention group patients were discharged to a geriatric rehabilitation facility (univariate odds ratio [OR] 1.97, 95% CI 1.1–3.54, *p* = 0.02; multivariate OR 2.02 95%CI 1.11–3.66, *p* = 0.02). Patients discharged to a rehabilitation facility had a longer average LOS due to the waiting time for transfer. The other secondary outcomes were not significantly different (Tab. 3). Patients discharged to a rehabilitation facility were generally admitted longer than the average patient population.

**Table 3:** Secondary outcomes compared between intervention group and control group

				Univariate analysis			Multivariate analysis	
	Control group *n* = 542	Intervention group *n* = 621	Effect size	95% CI	*p*-value	Effect size	95% CI	*p*-value
LOS in days median (range)	6 (72)	5 (51)	−0.08^†^		0.01*	−0.11^¶^	−0.21–−0.003	0.04*
In-hospital mortality *n* = 1151 *n* (%)	60 (11.0)	55 (8.9)	0.78^‡^	0.53–1.15	0.21	0.82	0.55–1.22	0.34
3‑month mortality *n* = 1151 *n* (%)	120 (22.3)	125 (20.4)	0.90^‡^	0.68–1.19	0.45	0.93	0.6–1.24	0.61
Change in residence situation *n* = 1048 *n* (%)	49 (10.2)	64 (11.3)	1.13^‡^	0.76–1.67	0.55	1.10	0.72–1.67	0.65
Discharge to geriatric rehabilitation *n* = 1048 *n* (%)	17 (3.5)	38 (7.2)	1.97^‡^	1.10–3.54	0.02*	2.02^§^	1.11–3.66	0.02*
Number of fallers *n* (%)	10 (1.8)	12 (1.9)	1.14^‡^	0.50–2.62	0.76	1.19	0.51–2.76	0.69
Patients with delirium *n* (%)	57 (10.5)	68 (11.0)	1.05^‡^	0.72–1.52	0.81	1.02	0.70–1.50	0.90
Number of consults mean (SD)	0.3 (0.6)	0.34(0.75)	−0.004^†^		0.88	0.98**	0.03–1.18	0.81

In all respective multivariate statistic models the covariates age, Charlson Comorbidity Index and history of chronic heart disease were included

Abbreviations: * *p* < 0.05, † Effect size r, ‡ logistic regression, § multiple logistic regression, ¶ unstandardized coefficient (B) from a multiple linear regression model, ** multivariate Poisson regression model

## Discussion

We found that after implementation of standard geriatric co-management the LOS decreased by 1 day (5 vs 6 days) and that more patients were discharged to geriatric rehabilitation facilities than before implementation (7.2% vs 3.5%). We found no difference in readmission rates, mortality, and occurrence of complications such as falls or delirium during admission.

### Hospital readmission

The hospital readmission rate was not significantly different before and after implementation of geriatric co-management, which is consistent with earlier findings [11–13]. In contrast, transitional care programmes with pre-discharge, bridging (phoned patient handover from secondary to primary care sector), and post-discharge components (home visits or follow-up calls) reduced the number of unplanned readmissions in older patients [21, 22]. Our study and the above-mentioned studies investigated pre-discharge interventions only. Patient handover or follow-up care might be needed to reduce the number of readmissions.

### Hospital length of stay

The decrease in LOS is an important finding, and confirms findings from previous research [12]. This is an important goal for improving patient health (reducing the risk of sarcopenia, functional decline, and nosocomial infections) and also from a socioeconomic perspective (decreasing healthcare costs).

The co-management intervention might have reduced the LOS by identifying potential health issues sooner—the geriatric team was involved in patient care within 24–48 h of admission, whereas the time to request a consult from a geriatrician is often longer. Thus delirium might be prevented instead of requiring treatment, or appropriate home care can be requested in time for patient discharge.

### Rehabilitation

We also found that the intervention increased the number of patients discharged to geriatric rehabilitation facilities. This illustrates a greater attention and effort of management of functional decline in the elderly. In other research this outcome was not analysed. However, co-managed patients have been shown to have better functional outcomes than usual-care patients [12, 13].

### Strengths and limitations

This is the first study to investigate the effect of geriatric co-management on important health outcomes in a large population of cardiology patients aged 85 and over. A multifactorial intention-to-treat analysis was used to correct for possible confounding. The project proved successful in increasing the number of frail elderly patients admitted to a cardiology ward who received geriatric care (from 14 to 64%). This study had few exclusion criteria and was nested in a real-world quality of care improvement programme. Therefore, the reproducibility of our results in regular practice is high.

Two factors might have led to underestimation of the effect of geriatric co-management. First, we did not screen patients using a frailty screening tool. This resulted in a heterogeneous subject population with resilient, pre-frail, and frail patients. Resilient patients might have benefited less from co-management, which could lead to an underestimation of the effect of the intervention. Second, through knowledge contamination [23], the detection and documentation of complications, such as delirium or falls, was probably increased during the intervention period in comparison with the control period. However, the intervention also reduced these complications during the intervention period. Ultimately, if both effects were of similar strength, then one would see no difference before and after the intervention.

Some limitations should also be considered in the interpretation of our results. First, the study design, a retrospective cohort design, is susceptible to confounding. We found a baseline difference in history of chronic heart disease and addressed this by adjusting for this in our multivariate analyses. However, there might have been residual confounding that could not be corrected. Second, we could not include outcomes relating to functional status or a cost-effectiveness analysis because of the retrospective study design.

### Recommendations for future research

The current findings suggest that geriatric co-management is an effective strategy in a multidisciplinary care model for older cardiology patients. The efficacy of geriatric co-management has previously been established in orthogeriatric patients with hip fractures and is emerging as standard of care [12]. Before geriatric co-management for older cardiology patients is implemented in routine clinical practice, more research is needed to investigate which elements of geriatric co-management are most effective in this population, and how to best identify those patients who might benefit from the intervention. This will also help in easing the logistics of starting future collaborative initiatives in regular practice.

## Conclusion

This study showed that geriatric co-management might be a viable strategy to optimise care for older hospitalised patients with cardiac disease, reducing the LOS and increasing the number of patients discharged to a geriatric rehabilitation facility. Further research is needed to establish the effectiveness of this intervention before it is implemented in daily practice.
